# Supplementary material for: The Patient’s Point of View: COVID-19 and Neuroendocrine Tumor Disease
Source: Cancers (Basel). 2022 Jan 26;14(3):613. doi: 10.3390/cancers14030613 (PMC8833713; doi:10.3390/cancers14030613)
Supplement: Supplementary file 1 [file cancers-14-00613-s001.zip › cancers-1514135-supplementary.pdf]

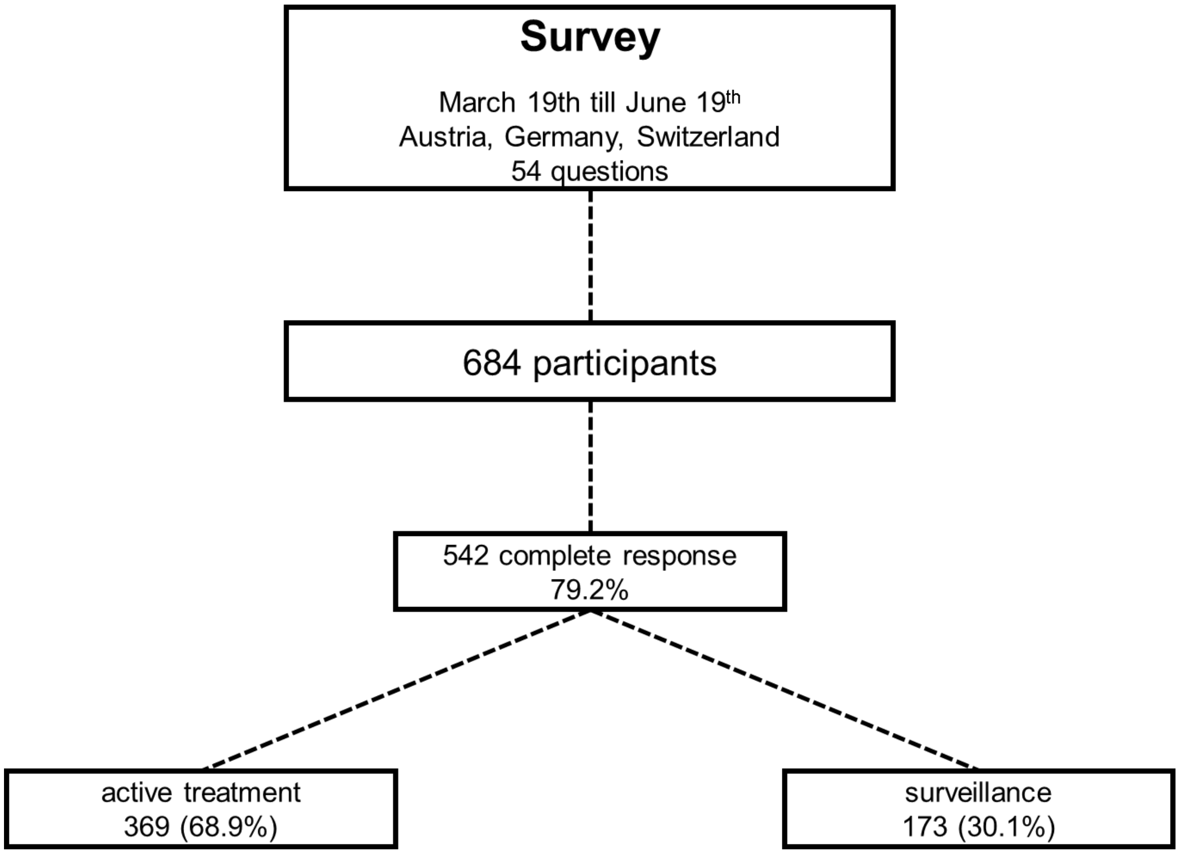

Figure S1. Flow chart of the survey.

Table S1. Complete questionnaire.

A1	In which country are you treated?
A2	Are you single, married, widowed or divorced?
A3	How old are you?
A4	Which NET disease did you or do you have?
A5	Is your disease hormone-active?
A6	Which hormone syndrome do you have?
A7	How long has your disease been known?
A8	How long was the time period between symptoms and diagnosis?
A9	Does the disease cause you any complaints?
A10	What are the complaints?
A11	Are you unable to work as a result of the disease?
A12	Have you been unable to go to work because of your disease IN THE LAST 3 MONTHS?
A13	In which facility are you treated or are you in surveillance?
A14	Are you undergoing oncological follow-up or active treatment?
A15	Which treatment are you currently receiving?
A16	Are you suffering from side effects of your current therapy?
A17	Which kind of side effects are present?
A18	Are you satisfied with the following points: medical treatment/therapy, organization and medical care
A19	Do you have any comorbidities?
S1	Are you worried about the Covid-19 pandemic?
S2	Has the Covid-19 pandemic affected your treatment or controls?
S3	In which area have your appointments been delayed or even had to be cancelled? (outpatient and inpatient appointments)
S3	In which area have your appointments been delayed or even had to be cancelled? (imaging MRI/CT, PET/CT, PRRT, surgical procedures, supportive therapies, treatment such as chemotherapy and patient organization meetings)
S4	Have you postponed/cancelled any treatments or follow-up appointments due to fear of Covid-19? (outpatient and inpatient appointments)
S5	Are you worried about going to hospital or to your consultation for treatment because of COVID-19? (outpatient and inpatient appointments)
S6	Have you used the option of telephone/video consultations?
S7	Have you talked to your NET specialist about your risk of severe COVID-19 disease?
S8	Has your physician recommended interventions to reduce your risk of acquiring COVID-19?
S9	Have you cancelled private or professional appointments (beyond the prescribed amount) to reduce the risk of acquiring Covid-19?
S10	Would you describe yourself as a risk group for severe Covid-19 disease?
S11	Do you feel isolated with your disease due to the Covid 19 pandemic?
S12	Do they have help with everyday shopping and general errands from family, friends and neighbours?

S13	Have you used support from a self-help group so far?
S14	Have you contacted a self-help group because of the Covid-19 pandemic?
S15	Have you searched/found specific information on the web about Covid-19 in relation to NET?
S16	Has the Covid-19 situation deteriorated your NET treatment?
S17	If no: Are you worried that the Covid-19 situation could lead to a deterioration of your NET care?
S18	What interventions would you like to see to improve care/treatment in the context of the Covid-19 pandemic?
S19	Has your treatment been modified as a result of the Covid-19 pandemic?
S20	Has your physician discussed with you the risk of severe Covid-19 disease during your current / intended therapy?
S21	Is there a difference in the accessibility of your physician before and now during the pandemic?
S22	Have you refrained from using public transport to get to the physician/hospital for fear of getting infected with Covid-19?
S23	Do you often wear an FFP2 mask, or always a "normal" mouth-nose protection (MNS)?
S24	Have you been tested for Covid-19?
S25	Did you have a Covid-19 infection?
S26	What was the course of the disease?
S27	Have you suffered or are you currently suffering from any after-effects of Covid-19?
S28	Did anyone in your social environment suffer from Covid-19?
S29	Would you get vaccinated against Covid-19?
S30	Can you explain why you refuse vaccination?
S31	Have you had the flu vaccination at least once in the last 5 years?
S32	Do you suffer from a psychiatric comorbidity in addition to your NET disease? (e.g. depression, anxiety disorder, unstable emotional personality disorder)
S33	If you answered 'yes' to the previous question: do you feel that the COVID-19 pandemic and its effects have had a negative impact on your psychological comorbidity?
S34	Please use the scale for the following statements.<br>Have you noticed an increase in the following symptoms since the start of the Covid-19 pandemic? Lack of happiness, loss of interest, sad mood, reduced motivation. Did you suffer from insomnia? Have you often felt socially isolated/lonely?
S35	Has the Covid-19 pandemic affected your work situation?
